# Pyloric Incompetence Associated with *Helicobactor pylori* Infection and Correlated to the Severity of Atrophic Gastritis

**DOI:** 10.3390/diagnostics12030572

**Published:** 2022-02-23

**Authors:** Takuki Sakaguchi, Takaaki Sugihara, Ken Ohnita, Daisuke Fukuda, Tetsuro Honda, Ryohei Ogihara, Hiroki Kurumi, Kazuo Yashima, Hajime Isomoto

**Affiliations:** 1 Department of Gastroenterology and Nephrology, Faculty of Medicine, Tottori University, Yonago 683-8504, Japan; sakagu-taku@tottori-u.ac.jp (T.S.); d21m1009h@edu.tottori-u.ac.jp (R.O.); kurumi_1022_1107@tottori-u.ac.jp (H.K.); yashima@tottori-u.ac.jp (K.Y.); 2 Department of Gastroenterology, Nagasaki University Hospital, Nagasaki 852-8501, Japan; k-ohnita@shunkaikai.jp (K.O.); dfukuda@fukudayutaka-geka.or.jp (D.F.); 3 Shunkaikai Inoue Hospital, Nagasaki 850-0045, Japan; 4 Department of Surgical Oncology, Graduate School of Biological Sciences, Nagasaki University, Nagasaki 852-8501, Japan; 5 Fukuda Yutaka Clinic, Nagasaki 852-8107, Japan; 6 Department of Gastroenterology, Nagasaki Harbor Medical Center, Nagasaki 850-8555, Japan; tetsupeacevo@yahoo.co.jp

**Keywords:** duodenogastric reflux, bile reflux gastritis, *Helicobacter pylori*, atrophic gastritis, pylorus

## Abstract

Duodenogastric reflux (DGR) causes bile reflux gastritis (BRG) and may develop into gastric cancer. DGR is classified as primary in non-operated stomachs or secondary to surgical intervention. Primary DGR and *Helicobacter pylori* (*H. pylori*) infection are reportedly related. However, the mechanism is not fully understood. This study aimed to elucidate the relationship between *H. pylori* infection and pyloric incompetence in a non-operated stomach. A total of 502 non-operated participants who underwent an upper intestinal endoscopy were prospectively enrolled. Endoscopic findings (EAC, endoscopic atrophy classification; nodular gastritis; xanthoma; fundic gland polyp; and incompetence of pylorus), sex, age, gastrin, pepsinogen (PG) I and PG II levels were evaluated. PG I/PG II ratio, anti-*H. pylori*-Ab positivity, and atrophic gastritis status were significantly different between the normal and incompetent pylori (*p* = 0.043, <0.001, and 0.001, respectively). Open-type atrophic gastritis was significantly higher in the incompetent pylori. Incompetence of the pylorus and EAC were moderately correlated (Cramer’s V = 0.25). Multivariate analysis revealed that the presence of anti-*H. pylori*-Ab was the only independent factor associated with the incompetence of the pylorus, with an adjusted odds ratio of 2.70 (95% CI: 1.47–4.94, *p* = 0.001). In conclusion, pyloric incompetence was associated with *H. pylori* infection and moderately correlated to the severity of atrophic gastritis in non-operated stomachs.

## 1. Introduction

Among reflux diseases in the gastrointestinal (GI) tract, the competence of the junctions is critical. Incompetence of the esophagogastric sphincter is well known to cause gastroesophageal reflux disease (GERD) [1]. The next junction is the pylorus. Duodenogastric reflux (DGR) is the passing of the duodenal contents through the pylorus into the stomach [2]. DGR is a physiological phenomenon that occurs in the early morning, postprandial periods, and during endoscopy and is also common after cholecystectomy, pyloroplasty, and gastric surgery [2,3,4,5]. Bile reflux gastropathy (BRG) or bile gastropathy (BG) is a pathological condition of DGR [6,7]. BRG accounts for approximately 22.6% of chronic gastritis [8]. Long-term DGR has already been known to cause pathological conditions, such as chronic gastritis, foveolar hyperplasia, intestinal metaplasia, gastric dysplasia, and gastric cancer [9,10,11,12,13,14]. DGR and BRG are classified into either (i) Primary or (ii) Secondary. Those classified as Primary are those that did not receive surgical interventions [15]. Primary DGR is considered a rare disease entity [16,17]. In the 1960s, it was reported that bile acid impaired the gastric mucosa and may be related to gastric ulcers; however, this remained unclear [18,19]. In the 1970s, researchers enthusiastically investigated the cause of DGR [16,20,21,22,23,24], wherein pyloric incompetence was indicated as a possibility [20]. In primary DGR/BRG, gastroduodenal ulcers were proposed as one of the factors for pyloric incompetence [16]. The mechanism of gastric ulcers diminishing the competence of the pylorus is explained as decreasing antral motility in the presence of gastric ulcers [20,25]. However, gastric ulcers may also be secondary to DGR, posing the “chicken-or-egg question”. Cocking et al. [16] discussed this in their manuscript and proposed smoking as another presumable primary cause of DGR. Read et al. demonstrated that the reflux occurred within 30 s from the time that an individual would start to smoke, as viewed in most cases of 13 normal volunteers [22]. It seemed that a consensus had been reached regarding the cause of DGR.

After Warren and Marshall’s first report of *Helicobacter pylori* (*H. pylori*) and chronic gastritis in 1983 [26], some investigators reported the association between DGR and *H. pylori* in the 1990s [8]. Ladas et al. indicated that DGR is consistently reduced after successful *H. pylori* eradication [8]. It implies that pyloric incompetence may be affected by the presence of an *H. pylori* infection. However, the relationship has still been controversial [27,28]. The causative mechanism of *H.* *pylori* infection for DGR remains unclear. In this study, we aimed to elucidate the relationship between *H. pylori* infection and pyloric incompetence in a non-operated stomach.

## 2. Materials and Methods

### 2.1. Study Design and Protocols

This study was a single-center, prospective, observational study conducted between August and December 2013 in Yutaka Fukuda Clinic (Nagasaki, Japan). The inclusion criteria were as follows: (i) participants who underwent upper gastrointestinal endoscopy for healthy check-up without any antispastics, including scopolamine butylbromide and glucagon, and underwent blood tests; (ii) those who were under 80 years of age; and (iii) those without a history of surgery for the stomach and *H. pylori* eradication therapy. They did not have any particular symptoms.

**Figure 1:**
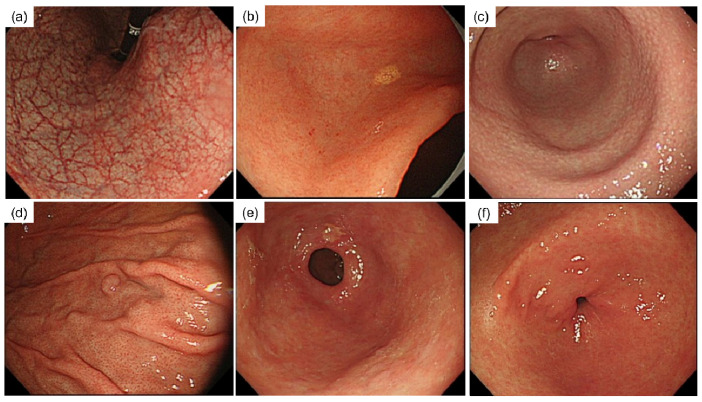
Essential endoscopic findings. (**a**) Endoscopic atrophic gastritis (Open type); (**b**) xanthoma; (**c**) nodular gastritis; (**d**) fundic gland polyp; (**e**) incompetent pylorus; (**f**) normal pylorus.

Finally, 502 participants (217 men and 285 women, median age 53 [23–77] years old) were continuously enrolled. One expert endoscopist, who did not receive the blood test results, assessed the following items: endoscopic atrophic degree, nodular gastritis, xanthoma, fundic gland polyp, and pyloric incompetence. The endoscopic atrophy classification (EAC) was assessed using the Kimura and Takemoto classification [29]. We defined “endoscopic pyloric incompetence” as the absence of resistance from the endoscope (GIF-PQ260; Olympus, Tokyo, Japan) as it traversed the pylorus (Figure 1).

The blood test was conducted on the same day as upper gastroenteroscopy following overnight fasting. The serum anti-*H. pylori* immunoglobulin G antibody (anti-*H. pylori*-Ab) was measured using an enzyme immunoassay kit using antigens derived from Japanese individuals (E-plate Eiken *H. pylori* antibody II; Eiken Chemical, Tokyo, Japan); the serum levels of pepsinogen I and II (PG I, II) and gastrin were measured using chemiluminescent enzyme radioimmunoassay (Eiken Chemical) and radioimmunoassay kit (Fujirebio, Tokyo, Japan), respectively. An *H. pylori* antibody titer of ≥10 U/mL was classified as *H. pylori*-positive [30]. The value of PG I/PG II was calculated as values of PG I levels divided those of PG II levels. We decided the cutoff values for PG I, PG I/PG II ratio, and gastrin as 70 ng/mL, 3, and 150 pg/mL, respectively [31,32]. We performed uni- and multivariate analyses to examine the correlations between pyloric incompetence and other endoscopic findings.

### 2.2. Statistical Analysis

Welch’s *t*-test and chi-squared test were applied to compare the two groups. The logistic regression model was applied for multivariate analyses. The adjusted odds ratio (OR) with a 95% confidence interval (CI) was also calculated. The Cochran–Armitage test and Cramer’s V were applied to examine the correlation between two categorical variables. The sensitivity, specificity, positive predictive value (PPV), and negative predictive value (NPV) were also investigated. All statistical analyses were performed using R (Ver. 4.0.3) or StatFlex (Windows ver. 6.0; Artech, Osaka, Japan). Values were expressed as mean with standard deviation (SD). Statistical significance was set at *p* < 0.05.

## 3. Results

### 3.1. Baseline Characteristics of the Participants

Table 1 shows the basic characteristics of all participants. The average age of the participants was 53.0 ± 10.9 years, and 217 (43.2%) participants were male. A total of 110 (21.9%) participants had open-type atrophic gastritis. A total of 200 (39.8%) participants were *H. pylori*-positive, and 55 (11.0%) participants’ pylori were deemed as endoscopically incompetent.

### 3.2. Comparison of Factors according to the Competency of the Pylorus

We first divided the participants into incompetent pylorus (n = 55) and normal pylorus (n = 447) groups. The age, serum gastrin level, PG I and PG II levels, PG I/PG II, and endoscopic atrophic classification were evaluated. Endoscopic atrophic gastritis was divided into closed type or open type by EAC. Open-type atrophic gastritis was observed in 87 (19.5%) and 23 (41.8%) participants in the normal and incompetent pylorus groups, respectively.

We then conducted the univariate analysis on the baseline characteristics and other endoscopic findings between the incompetent pylorus group and in all participants. We found that the PG I/PG II levels (4.4 ± 1.8 vs. 3.8 ± 1.9, *p* = 0.043), participants whose anti-*H. pylori*-Ab was positive (37.1% vs. 61.8%, *p* < 0.001), and participants who had atrophic gastritis (80.1% vs. 98.2%, *p* = 0.001) were significantly different between the incompetent pylorus and the normal pylorus group (Table 2).

**Figure 2:**
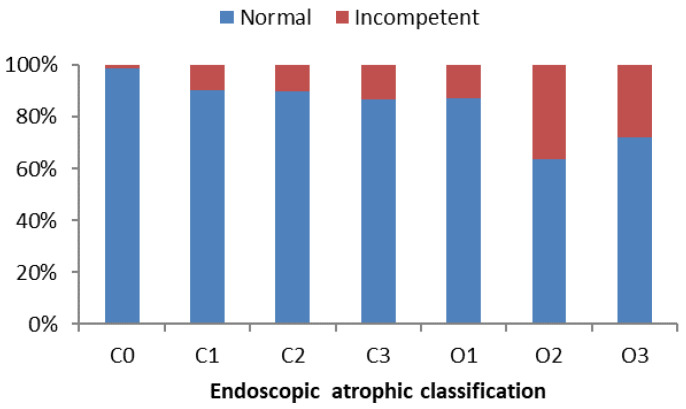
Endoscopic atrophic classification and competence of pylorus. The incidence of pyloric incompetence increased with the severity of atrophic gastritis. Particularly, the incidence of incompetent pylori was significantly higher in open-type atrophic gastritis (8.2% vs. 20.9%, *p* < 0.001).

Furthermore, we analyzed EAC between the two groups. The presence of incompetent pylori was higher with open-type atrophic gastritis (O-I, -II, -III) (8.2% vs. 20.9%, *p* < 0.001) (Figure 2). There was a moderate relationship between incompetent pylori and EAC (Cramer’s V = 0.25). The presence of open-type atrophic gastritis was significantly higher in the anti-*H. pylori*-Ab positive group (14.35 vs. 58.8%, *p* < 0.001) and strongly-related (Cramer’s V = 0.73) to the incompetent pylorus group.

Thereafter, we conducted a multivariate analysis to distinguish independent factors related to pyloric incompetence from other confounding factors and calculated the adjusted odds ratio (OR) with 95% CI (Table 3). PG I/PG II ratio < 3 and EAC were strongly related to anti-*H. pylori*-Ab positivity (Cramer’s V = 0.65 and 0.65, respectively); therefore, we excluded these factors. The presence of anti-*H. pylori* antibodies was the only independent factor associated with pyloric incompetence, with an adjusted odds ratio of 2.70 (95% CI: 1.47–4.94, *p* = 0.001).

### 3.3. Incompetence of the Pylorus as a Predictive Endoscopic Finding for H. pylori Infection

Furthermore, we also evaluated whether pyloric incompetence can be a predicting endoscopic finding for *H. pylori* infection. The sensitivity, specificity, positive predictive value (PPV), and negative predictive value (NPV) were calculated for predicting *H. pylori* infection in known predictive endoscopic findings (nodular gastritis, xanthoma, and absence of fundic gland polyp) and pyloric incompetence (Table 4). The sensitivity of pyloric incompetence was higher than the others, while its specificity was the lowest. Although PPV was the lowest among the endoscopic findings, NPV was similar to xanthoma and the absence of fundic gland polyps.

## 4. Discussion

In this study, we demonstrated that *H. pylori* infection was an independent factor for pyloric incompetence in non-operated stomachs. Moreover, the severity of atrophic gastritis was moderately related to the incompetence of the pylorus. 

Some of the earliest reports indicated that bile reflux caused a gradual elimination of *H. pylori* in vitro or in the stomach postoperatively [33,34]. However, later, Ladas et al. [8] indicated that the infection might induce DGR in a non-operated stomach, and discussed that the difference was due to the lower bile acid concentrations when compared to operated stomachs. 

Matsuhisa et al. [35] reported that *H. pylori* infection status did not affect the risk of atrophic gastritis in DGR. However, in our study, the anti-*H. pylori*-Ab positivity rate among participants with incompetent pylori was significantly high (61.8%). Indeed, endoscopic pyloric incompetence is not diagnostic of DGR; however they are considered to be closely associated [20]. Moreover, the incidence of open-type atrophic gastritis was significantly higher in the anti-*H. pylori*-Ab positive group and had a strong correlation to the incompetent pylorus group. Atrophic gastritis is the first step in Correa’s cascade [36]. Pathologically, original gastric glands are replaced by intestinal metaplasia and pseudo-pyloric metaplasia, with or without fibrosis [37]. Li et al. [27] demonstrated that intestinal metaplasia was most prevalent among patients with *H. pylori* infection and a high bile acid concentration in the gastric juice. These data suggest that both *H. pylori* infection and the resulting atrophic gastritis may affect the competence of the pylorus.

We hypothesized that *H. pylori* infection induces open-type atrophic gastritis, wherein severe atrophy impairs the function of the pylorus; the incompetent pylorus consequently causes DGR. 

Interestingly, Szőke et al. demonstrated that smoking is associated with *H. pylori* infection in patients with DGR [28]. Smoking has already been proposed as a risk factor for pyloric incompetence [16,22]. Smoking was reported to cause an imbalance in the antioxidant equilibrium and increase the risk of *H. pylori* infection [38]. We did not evaluate smoking habits in this study; however, according to the reports in the 1970s, smoking has undoubtedly accelerated the incompetence of the pylorus in combination with *H. pylori* infection. Our study findings may have partially fulfilled the goals of the 50-year investigations on the mechanism of primary DGR/BRG. 

It is estimated that 0.1% to 0.3% of atrophic gastritis cases develop into gastric adenocarcinomas annually [36]. Patients diagnosed with open-type endoscopic atrophy have a higher risk of gastric cancer development than those diagnosed with closed-type [39,40,41]. Chronic *H. pylori* infection is a leading cause of gastric cancer [42,43]. The combination of bile reflux and *H. pylori* infection may cause progression to gastric cancer that may not be through the traditional process: inflammation–atrophy–metaplasia [44]

Although the prevalence of *H. pylori* is decreasing, especially in developed countries with the expanded indication of *H. Pylori* eradication therapy, it is estimated that approximately 4.4 billion people are still infected with *H. pylori* worldwide [45]. Therefore, it is crucial to recognize patients with *H. pylori* infection and diagnose the severity of atrophic gastritis.

The Kyoto classification of gastritis was advocated at the 85th Congress of the Japan Gastroenterological Endoscopy Society to standardize endoscopic findings of gastritis. The Kyoto classification included categories of xanthoma, nodularity, and fundic gland polyp [39,46]. However, there is no description on the incompetence of the pylorus.

Our data demonstrated that the severity of atrophic gastritis was moderately related to pyloric incompetence. Therefore, we also investigated the sensitivity and specificity of pyloric incompetence for *H. pylori* infection. The sensitivity of pyloric incompetence was higher than those of nodular gastritis, xanthoma, and fundic gland polyp in predicting *H. pylori* infection. On the other hand, its specificity was lower. Incompetence of the pylorus may be a good predictor of *H. pylori* infections.

This study has several limitations. First, this is a single-center study, which enrolled only a small number of participants. Second, the study might have included participants who had unintentionally undergone *H. pylori* eradication therapy at another hospital or may have experience natural resolution of the infection. Third, there might be false-negative cases in diagnosing *H. pylori* infection with serum anti-*H. pylori*-Ab. It was reported that in a population with no history of eradication therapy, 17% of participants whose anti-*H. pylori*-Ab titer was 3–9.9 U/mL were false negative [30]. Fourth, we did not evaluate the bile acid in the stomach. However, we believe that we could partly elucidate the close relationship between *H. pylori* infection and incompetency of the pylorus.

In this study, we focused on the competence of the pylorus. In future studies, we may also focus on the last junction in the GI tract, which is the ileocecal valve; its incompetency causes “backwash ileitis” in ulcerative colitis [47]. It can also be called colon-ileal reflux disease (CIRD). GERD, DGR, and CIRD may be summarized as gastrointestinal reflux diseases (GIRD) in the future. In a broad sense, the incompetence of Vater’s papilla may also be included in GIRD. Further investigation is warranted for establishing this new concept.

## 5. Conclusions

In this study, we elucidated that the incompetence of the pylorus is associated with *H. pylori* infection and moderately correlates to the severity of atrophic gastritis in non-operated stomachs. Pyloric incompetence can also be a predictive endoscopic finding of *H. pylori* infection.

## Figures and Tables

**Table 1:** Basic characteristics of the participants.

n = 502		
Age (yr)	53.0 ± 10.9	
Male Sex	217	(43.2)
PG I (ng/mL)	54.50	(39.4)
PG II (ng/mL)	15.25	(11.4)
Serum gastrin * (pg/mL)	158.21 ± 100.34	
PG I/PG II ratio	4.31 ± 1.84	
anti-*H. pylori*-Ab positivity	200	(39.8)
Endoscopic atrophy classification		
C-0	90	(17.9)
C-I	163	(32.5)
C-II	101	(20.1)
C-III	38	(7.6)
O-I	63	(12.5)
O-II	22	(4.4)
O-III	25	(5.0)
Nodular gastritis	11	(2.2)
Incompetent pylorus	55	(11.0)

PG, prostaglandin. Ant-*H. pylori*-Ab, anti-*H. pylori* antibody. * Serum gastrin values were missing for 32 participants. Data are expressed as mean ± SD. Numbers in parentheses refer to the percentage of participants.

**Table 2:** Univariate analysis of factors according to the competency of the pylorus.

	Pylorus		*p*-Value
	Normal (n = 447)	Incompetent (n = 55)	
Male	192 (43.0)	25 (45.5)	0.834
Age (yr),	52.8 ± 10.8	54.8 ± 11.3	0.214
Serum gastrin * (pg/mL)	157.3 ± 97.7	165.2 ± 120.2	0.647
PG I (ng/mL)	55.3 ± 40.9	48.0 ± 23.5	0.054
PG II (ng/mL)	15.3 (11.7)	14.7 ± 7.8	0.612
PG I/PG II ratio	4.4 ± 1.8	3.8 ± 1.9	**0.043**
anti-*H. pylori*-Ab positivity	166 (37.1)	34 (61.8)	**<0.001**
Atrophic gastritis **	358 (80.1)	54 (98.2)	**0.001**
Nodular_gastritis	10 (2.2)	1 (1.8)	1.000
Xanthoma	21 (4.7)	6 (10.9)	0.054

* Serum gastrin values were missing for 32 participants. ** C-I to O-III by endoscopic atrophy classification were considered as atrophic gastritis. Data are expressed as mean ± SD. Numbers in parentheses refer to the percentage of participants. *p*-values indicative of a significant result are presented in bold print.

**Table 3:** Multivariate analysis of factors according to the competency of the pylorus.

	β *	SE	z Value	Odds Ratio	95% CI	*p*-Value
Age > 53	0.05	0.29	0.34	0.91	0.51–1.60	0.733
Male Sex	−0.10	0.31	0.17	1.05	0.58–1.93	0.868
anti-*H. pylori*-Ab positivity	0.99	0.31	3.22	2.70	1.47–4.94	**0.001**

* Estimated unstandardized regression coefficients. SE, standard error; CI, confidence interval; *p*-values indicative of a significant result are presented in bold print.

**Table 4:** Predictivity of factors for *H. pylori* infection.

	Sensitivity	Specificity	PPV	NPV
NG	4.3	99.8	81.8	58.9
Xanthoma	11.4	99.0	88.9	60.6
FGP absence	16.5	97.6	90.6	60.6
IP	16.6	93.1	63.6	60.6

NG, nodular gastritis; FGP, fundic gastric polyp; IP, incompetence of pylorus.

## Data Availability

Not applicable.
